# Sexual selection for extreme physical performance in a polygynous bird is associated with exceptional sex differences in oxygen carrying capacity

**DOI:** 10.1098/rsbl.2023.0391

**Published:** 2023-11-22

**Authors:** Peter Santema, Luke Eberhart-Hertel, Mihai Valcu, Bart Kempenaers

**Affiliations:** ^1^ Department of Ornithology, Max Planck Institute for Biological Intelligence, Seewiesen 82319, Germany; ^2^ Edward Grey Institute, Department of Biology, University of Oxford, Oxford, UK

**Keywords:** haematocrit, oxygen carrying capacity, pectoral sandpiper, polygyny, sexual selection

## Abstract

In many animals, males compete for access to fertile females. The resulting sexual selection leads to sex differences in morphology and behaviour, but may also have consequences for physiology. Pectoral sandpipers are an arctic-breeding polygynous shorebird in which males perform elaborate displays around-the-clock and move over long distances to sample potential breeding sites, implying the need for physiological adaptations to cope with extreme endurance. We examined the oxygen carrying capacity of pectoral sandpipers, measured as the volume percentage of red blood cells in blood (haematocrit, Hct). We found a remarkable sex difference in Hct levels, with males having much higher values (58.9 ± 3.8 s.d.) than females (49.8 ± 5.3 s.d.). While Hct values of male pectoral sandpipers are notable for being among the highest recorded in birds, the sex difference we report is unprecedented and more than double that of any previously described. We also show that Hct values declined after arrival to the breeding grounds in females, but not in males, suggesting that males maintain an aerobic capacity during the mating period equivalent to that sustained during trans-hemispheric migration. We conclude that sexual selection for extreme physical performance in male pectoral sandpipers has led to exceptional sex differences in oxygen carrying capacity.

## 1. Introduction

Sexual selection, i.e. the process whereby one sex (typically the male) competes for access to the other sex (typically the female), is the driving force behind the evolution of conspicuous secondary sexual characteristics [1,2]. This is exacerbated in polygynous species, in which a small proportion of males typically sires the majority of offspring [3]. Males in these species often develop distinct morphological traits, such as colourful ornamentation and imposing weaponry [4,5], and engage in elaborate behavioural displays to attract females and intimidate other males [6]. While the behavioural and morphological characteristics produced by sexual selection are well studied, the potential effects of sexual selection on physiological differences between the sexes has received much less attention [7,8].

When competition among males for access to females is intense and males with superior performance achieve the highest mating success, sexual selection should favour improved physical performance in males [9]. An essential aspect of an individual's physical performance is its oxygen carrying capacity, which determines the amount of oxygen circulated to organs and plays a key role in oxidative metabolism [10]. In wild vertebrates, oxygen carrying capacity is most commonly measured as haematocrit (Hct), which is the volume percentage of red blood cells in blood [10]. Hct measures are relatively easy to obtain, even from animals in the wild, and estimates of Hct levels from more than 270 species have been published to date in birds alone (reviewed in [11]). Hct levels can vary seasonally within an individual and rise when energetic demands increase, most notably during migration [12–16]. Intense intra-sexual competition between males during the breeding season may similarly lead to high Hct levels [8].

The pectoral sandpiper is a socially polygynous shorebird that winters in South America and migrates 7000–14 000 km to tundra breeding grounds in the Arctic [17]. Upon arrival at a breeding site from late May onwards, males set up display territories from which they aggressively exclude other males. Females start arriving a few days later and initiate nests soon thereafter, which they tend to alone. Under the 24 h daylight of the Arctic, competition among males for access to females is intense: they perform aerial displays and compete with other males around-the-clock, to the extent that they almost entirely dispose of sleep [18]. Moreover, when local mating opportunities diminish or are lacking, males visit other potential breeding sites, flying thousands of kilometres across much of the species' breeding range, encompassing the Russian and North American Arctic [17]. Competition for access to females has thus led to selection for extreme physical performance in male pectoral sandpipers.

We studied oxygen carrying capacity of pectoral sandpipers at a breeding site in the Arctic. Over nine breeding seasons, we collected a total of 1040 blood samples (778 male, 262 female) and measured Hct levels. First, we tested whether male and female pectoral sandpipers differed in their blood Hct levels. Second, we assessed how sex differences in pectoral sandpiper Hct values compared to sex difference estimates reported in other bird species. Third, we examined whether intrinsic and extrinsic factors explain variation in Hct levels between individuals.

## 2. Methods

### (a) Study site and general procedures

We studied pectoral sandpipers at an approximately 2 km^2^ site at the northern tip of the Arctic coastal plain near Utqiagvik, Alaska (71°18′ N, 156°44′ W) in the years 2004–2009, 2012, 2014 and 2018. We caught pectoral sandpipers during the breeding season using hand-held mist nets (males and females) or nest traps (females only). We assigned each bird a metal leg band and a unique combination of colour leg bands for individual identity. At each capture, we weighed them (to the nearest 0.1 g), measured their tarsus (to the nearest 0.1 mm), and sampled 200–300 µl of blood using brachial venepuncture. We collected blood in 70 µl heparinized microhaematocrit capillary tubes and centrifuged the samples at 5000 rpm for 10 min on the day of collection, thus separating plasma from cellular blood. Hct levels were measured for each full capillary as the percentage of packed red blood cells over the total blood sample. For statistical analyses, we used the mean value from all capillary tubes obtained from an individual during a given capture. Red blood cells were kept and stored in Queen's lysis buffer for subsequent molecular sexing. In total, we obtained 778 blood samples from males and 262 from females. For 38 males we obtained multiple samples within a breeding season (i.e. two (*N* = 34), three (*N* = 3), or four (*N* = 1)). Additionally, for eight males we obtained samples across two (*N* = 6) or three (*N* = 2) breeding seasons. We attempted to find all nests on the study site by (1) observing foraging females until they went to their nest to incubate or (2) flushing females off their nest by systematically searching or rope dragging the area.

### (b) Comparative data

We obtained estimates of Hct values from other bird species from Minias [11]. Minias [11] compiled a dataset of 611 Hct estimates from 279 species based on data available in the published literature. This dataset includes only non-experimental studies, and only studies on wild birds or birds kept in outdoor aviaries (i.e. studies on birds kept indoors were not included). From this dataset, we extracted all studies that reported a separate estimate for males and females from the same age class (juvenile or adult). We only included studies when the Hct estimate for each sex was based on at least 10 individuals, resulting in a dataset consisting of 63 estimates of male and female Hct values from 35 different species.

We additionally obtained sex-specific Hct estimates from five species at our study site which we had collected as part of parallel research projects. These samples were obtained and processed in the same way as those of pectoral sandpipers. Thus, we included data from American golden plover *Pluvialis dominica* (*n* = 10 males, 12 females), dunlin *Calidris alpina* (*n* = 10 males, 10 females), long-billed dowitcher *Limnodromus scolopaceus* (*n* = 26 males, 21 females), red phalarope *Phalaropus fulicarius* (*n* = 257 males, 312 females), and semipalmated sandpiper *Calidris pusilla* (*n* = 71 males, 67 females).

The combined set of data extracted from Minias [11] and collected by ourselves (excluding pectoral sandpipers) thus consisted of 68 estimates of male and female mean Hct values from 40 different species.

### (c) Statistical analyses

All statistical analyses were performed with R (version 4.2.2, www.r-project.org). We performed (generalized) linear mixed models using the lme4 package [19]. We used the R package ‘multcomp’ [20] to obtain *p*-values corrected for within-model multiple testing.

To test for a difference in Hct levels between male and female pectoral sandpipers, we performed a *t*-test. To test whether Hct levels in male and female pectoral sandpipers differed from that of males, respectively females, of other species (68 estimates from 40 species), we performed one sample *t*-tests. To test for a difference in average Hct levels between males and females across different bird species (68 male and female estimates from 40 species), we performed a paired *t*-test.

To examine factors associated with variation in Hct values in pectoral sandpipers, we performed linear mixed-effect models with Hct value as the response variable. Because factors affecting Hct levels may differ between the sexes, we ran a model for males and females separately. We included date, body mass (g) and tarsus length (mm) as fixed effects. For females, we also included breeding status, i.e. whether they nested locally at our study site during a given season, as well as the interaction effect of breeding status with date. Breeding status was not included in the model for males, because males mate opportunistically at different breeding sites and can all be considered potential breeders. Moreover, previous work showed that males who sired offspring locally did not differ in Hct values from males that did not [17]. Numeric variables were mean-centred, so that the model intercept was estimated for the mean value of the explanatory variables. Body mass and tarsus length correlated positively, but weakly (Pearson's correlation coefficients; males: *r* = 0.24, females: *r* = 0.13) and both were thus included as explanatory variables in the models. Year was included as a random effect in both models. Because we had more than one sample for some males, we also included individual identity as a random intercept in the model for males.

## 3. Results

### (a) Difference between males and females

**Figure 1.**
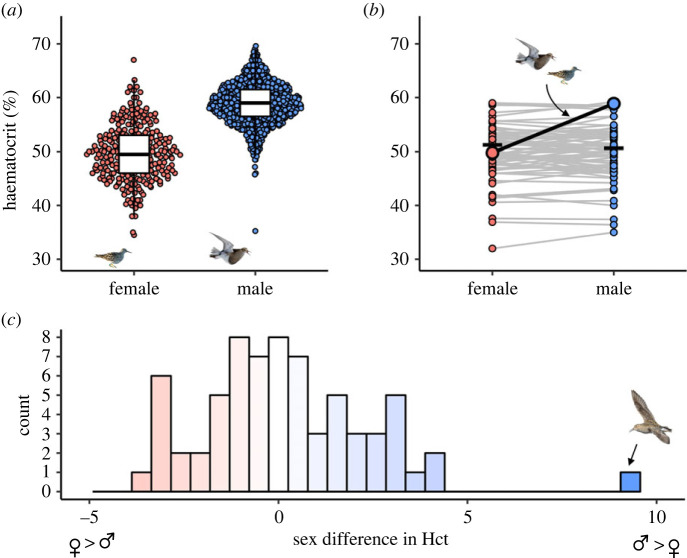
(*a*) Haematocrit levels of male and female pectoral sandpipers during the breeding season. Boxplots show the median, first and third quartile, and 1.5 × interquartile range. (*b*) Mean male and female Hct values in 40 bird species, including the pectoral sandpiper (black line). Lines connect the male and female from the same study, and dashes show the average values, weighted by sample size, across species (not including pectoral sandpiper). (*c*) Histogram of sex differences in Hct levels (percentage point) across bird species, including the pectoral sandpiper (arrow). Data from other bird species were obtained from the published literature (*N* = 63 estimates from 35 species) or collected by us (*N* = 5 species; see Methods).

The average Hct level in pectoral sandpipers was 58.9 (±3.8 s.d.) for males and 49.8 (±5.3 s.d.) for females (figure 1*a*). Hct values of males were thus on average 9.1 percentage points higher than those of females. This difference between males and female was highly statistically significant (*t* = 25.75, d.f. = 358.59, *p* < 0.001).

### (b) Between-species comparison of sex differences

Average Hct levels across 68 estimates from 40 different species (weighted by sample size) were 51.0 (±4.3 s.d.) for males and 51.2 (±4.4 s.d.) for females (figure 1*b*). Hct levels in male pectoral sandpipers were significantly higher than those in males of other species (one-sample *t*-test, *t* = −14.86, d.f. = 67, *p* > 0.001). However, Hct levels in female pectoral sandpipers did not differ from those in females of other species (one-sample *t*-test, *t* = −0.20, d.f. = 67, *p* = 0.84).

There was no overall sex difference in Hct levels across species (paired *t* = 0.20, d.f. = 67, *p* = 0.84). The sex difference in Hct levels reported in previous studies ranged from −3.6 percentage points (i.e. females > males, house sparrow *Passer domesticus*), to 4.3 percentage points (i.e. males > females, green-rumped parrotlet *Forpus passerinus*) (figure 1*c*). The sex difference in pectoral sandpipers (9.1 percentage points) is thus over twice that of any previously described avian sex difference in Hct values (figure 1*c*).

### (c) Correlates of Hct

**Figure 2.**
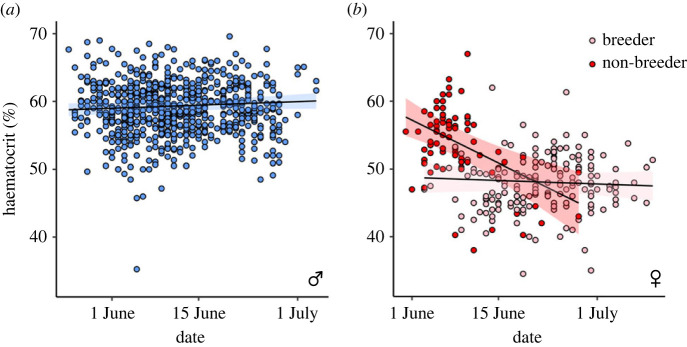
Changes in haematocrit (Hct) levels in male (*a*) and female (*b*) pectoral sandpipers over the breeding season. Points show raw data and lines and shaded areas show predictions (± 95% CI) based on the model described in table 1.

Date did not explain variation in Hct levels in male pectoral sandpipers (table 1, figure 2*a*). However, Hct values in males were strongly positively correlated with body mass, with Hct levels increasing by 1.7 percentage points for every 10 g gain in body mass (table 1). Hct levels in males were negatively associated with tarsus length, with a decrease in Hct levels of 0.5 percentage point for every 1 mm increase in tarsus length (table 1). Thus, structurally larger males had lower Hct values, but males that were heavier (i.e. accounting for their body size) had higher Hct values.

**Table 1.** Results of linear mixed models examining the effect of date, body mass, tarsus length, and for females only, breeding status and the interaction of breeding status with date on haematocrit levels in pectoral sandpipers (*N* = 778 males, 262 females). Numeric response variables were mean-centred.

	variable	estimate	s.e.	d.f.	*z*	*p*
males	(intercept)	58.16	0.39	10.06		
	date	0.03	0.02	309.79	1.65	0.24
	body mass	0.17	0.02	682.43	10.04	<0.001
	tarsus	−0.43	0.13	700.49	−3.26	0.003
females	(intercept)	47.49	1.04	25.75		
	date	−0.03	0.05	249.56	−0.55	0.98
	body mass	−0.04	0.03	250.36	−1.32	0.60
	tarsus	−0.34	0.25	251.69	−1.37	0.56
	breeding status^a^	2.92	0.83	244.75	3.53	0.002
	date × breeding status^a^	−0.42	0.10	246.58	−4.05	<0.001

^a^Non-breeder relative to breeder.

There was a significant interaction effect between breeding status and date on female Hct values (table 1, figure 2*b*). Non-breeding females had high Hct levels at the beginning of the breeding season, but their Hct levels declined over the course of the season (figure 2*b*). Females that bred at our study site had lower Hct levels that remained constant throughout the season (figure 2*b*). Body mass and tarsus length did not explain variation in Hct levels in females (table 1).

## 4. Discussion

We investigated oxygen carrying capacity in pectoral sandpipers by measuring their Hct levels on their Arctic breeding grounds. We found that males had much higher values than females (figure 1*a*): a sex difference more than twice that of any previously reported in a bird (figure 1*c*). The extreme mating system of pectoral sandpipers, in which males are alert, perform displays, fight with other males around-the-clock, and make long-distance flights between potential breeding sites, thus appears to have resulted in strong sexual dimorphism in aerobic capacity. This highlights the potential for sexual selection to shape physiological differences between the sexes, and underscores the importance of the mating system for the evolution of physiological traits. Although we emphasize selection on males for elevated Hct levels, factors constraining high Hct levels in females could also contribute to the extreme sex difference in this species. However, we note that the Hct levels in female pectoral sandpipers are similar to those in females of other species, while male pectoral sandpipers have exceptionally high Hct levels compared to males of other species (figure 1*b*).

Hct levels in non-breeding females that were caught early in the season were high, and similar to those of males (figure 2*b*). Most of these females were caught in an inundated swath of tundra that is typically used only for foraging and were never subsequently re-encountered. Hence, they likely used the study site as a staging area during migration to a breeding location elsewhere in the Arctic. Females thus appear to have high Hct levels during spring migration (a approximately 10 000 km journey from South America), but reduce Hct levels when arriving at their Arctic breeding grounds. Indeed, Hct values declined rapidly upon arrival at the breeding grounds in other migratory bird species [12,21]. By contrast, male pectoral sandpipers had consistently high Hct levels throughout the breeding season, and thus appear to maintain similar Hct levels during their tenure on the breeding grounds as during their migration (figure 2*a*). This suggests that their around-the-clock activity [18] and large-scale breeding site sampling [17] may require an aerobic capacity equivalent to that during their trans-hemispheric spring migration.

Comparative studies on birds indicate that males and females do not generally differ in Hct levels [11,22]. Our pairwise comparisons also suggest that sex differences in Hct levels are typically small, and that average Hct levels were about as likely to be higher in males (*N* = 31) as in females (*N* = 35) (figure 1*c*). However, a recent study that compared Hct values in males and females from 68 bird species sampled during the breeding season reported higher Hct levels in males [8]. Thus, higher Hct values in males compared to females may not be unique to pectoral sandpipers. Nevertheless, the sex difference in pectoral sandpipers was substantially larger than any of the sex differences reported in Vincze *et al*. [8]. Sex differences in Hct levels may only emerge during the mating period, which may be why previous comparative studies that used data collected during all stages of the annual cycle found no overall sex difference [11,22]. It would be worthwhile to investigate how sex differences in Hct levels change over the annual cycle, in pectoral sandpipers and in birds generally. A study on 45 primate species also found clear evidence for higher Hct levels in males than in females [7]. A possible explanation for this difference is that most primates, and indeed most mammals, are socially polygynous [23], and thus experience high levels of male–male competition [24]. Most bird species, in contrast, are socially monogamous [25], which may limit the scope for sexual selection and the evolution of sexually dimorphic physiology [26].

In conclusion, we show that the extreme mating behaviour of pectoral sandpipers is associated with exceptional sexually dimorphic physiology. Further work is needed to test whether variation in the degree of male–male competition and differences in physical performance between males and females can explain sex differences in oxygen carrying capacity across species. While the conspicuous effects of sexual selection on morphology and behaviour have, perhaps understandably, received the majority of research attention, our findings indicate that the effects of sexual selection on physiology may be equally striking and deserve more attention.

## Data Availability

The data used in this study are available from the Dryad Digital Repository: https://doi.org/10.5061/dryad.pzgmsbcss [27].

## References

[1] Darwin C. 1871 The descent of man, and selection in relation to sex, vol. 2. London, UK: John Murray.

[2] Andersson M. 1994 Sexual selection. Princeton, NJ: Princeton University Press.

[3] Wiley RH. 1991 Lekking in birds and mammals: behavioral and evolutionary issues. In Advances in the study of behavior, pp. 201-291. London, UK: Academic Press.

[4] Berglund A, Bisazza A, Pilastro A. 1996 Armaments and ornaments: an evolutionary explanation of traits of dual utility. Biol. J. Linn. Soc. **58**, 385-399. (10.1111/j.1095-8312.1996.tb01442.x)

[5] Dale J, Dey CJ, Delhey K, Kempenaers B, Valcu M. 2015 The effects of life history and sexual selection on male and female plumage colouration. Nature **527**, 367-370. (10.1038/nature15509) 26536112

[6] Searcy WA, Andersson M. 1986 Sexual selection and the evolution of song. Ann. Rev. Ecol. Syst. **17**, 507-533. (10.1146/annurev.es.17.110186.002451)

[7] Lindenfors P, Revell LJ, Nunn CL. 2010 Sexual dimorphism in primate aerobic capacity: a phylogenetic test. J. Evol. Biol. **23**, 1183-1194. (10.1111/j.1420-9101.2010.01983.x) 20406346 PMC2926801

[8] Vincze O, Vágási CI, Pénzes J, Szabó K, Magonyi NM, Czirják GÁ, Pap PL. 2022 Sexual dimorphism in immune function and oxidative physiology across birds: the role of sexual selection. Ecol. Lett. **25**, 958-970. (10.1111/ele.13973) 35106902 PMC9305230

[9] Byers J, Hebets E, Podos J. 2010 Female mate choice based upon male motor performance. Anim. Behav. **79**, 771-778. (10.1016/j.anbehav.2010.01.009)

[10] Campbell TW. 1995 Avian hematology and cytology. Ames, IA: Iowa State University Press.

[11] Minias P. 2020 Ecology and evolution of blood oxygen-carrying capacity in birds. Am. Nat. **195**, 788-801. (10.1086/707720) 32364788

[12] Morton ML. 1994 Haematocrits in montane sparrow in relation to reproductive schedule. Condor **96**, 117-126.

[13] Piersma T, Everaarts JM, Jukema J. 1996 Build-up of red blood cells in refuelling bar-tailed godwits in relation to individual migratory quality. Condor **98**, 363-370.

[14] Møller AP, Vágási CI, Pap PL. 2013 Risk-taking and the evolution of mechanisms for rapid escape from predators. J. Evol. Biol. **26**, 1143-1150. (10.1111/jeb.12147) 23617805

[15] Krause JS, Németh Z, Pérez JH, Chmura HE, Ramenofsky M, Wingfield JC. 2016 Annual hematocrit profiles in two subspecies of white-crowned sparrow: a migrant and a resident comparison. Physiol. Biochem. Zool. **89**, 51-60. (10.1086/684612) 27082524

[16] Yap KN, Tsai OH, Williams TD. 2019 Haematological traits co-vary with migratory status, altitude and energy expenditure: a phylogenetic, comparative analysis. Sci. Rep. **9**, 6351. (10.1038/s41598-019-42921-4) 31011157 PMC6476874

[17] Kempenaers B, Valcu M. 2017 Breeding site sampling across the Arctic by individual males of a polygynous shorebird. Nature **541**, 528-531. (10.1038/nature20813) 28068667

[18] Lesku JA, Rattenborg NC, Valcu M, Vyssotski AL, Kuhn S, Kuemmeth F, Heidrich W, Kempenaers B. 2012 Adaptive sleep loss in polygynous pectoral sandpipers. Science **337**, 1654-1658. (10.1126/science.1220939) 22878501

[19] Bates D, Mächler M, Bolker B, Walker S. 2014 Fitting linear mixed-effects models using lme4. J. Stat. Softw. **67**, 1-48.

[20] Hothorn T, Bretz F, Westfall P. 2008 Simultaneous inference in general parametric models. Biom. J. **50**, 346-363. (10.1002/bimj.200810425) 18481363

[21] Jones PJ. 1983 Haematocrit values of breeding redbilled queleas (*Quelea quelea*) (Aves: Ploceidae) in relation to body condition and thymus activity. J. Zool. **201**, 217-222. (10.1111/j.1469-7998.1983.tb04271.x)

[22] Fair J, Whitaker S, Pearson B. 2007 Sources of variation in haematocrit in birds. Ibis **149**, 535-552. (10.1111/j.1474-919X.2007.00680.x)

[23] Kappeler PM, van Schaik CP. 2002 Evolution of primate social systems. Int. J. Primatol. **23**, 707-740. (10.1023/A:1015520830318)

[24] Clutton-Brock T. 2021 Social evolution in mammals. Science **373**, eabc9699. (10.1126/science.abc9699) 34529471

[25] Cockburn A. 2006 Prevalence of different modes of parental care in birds. Proc. R. Soc. B. **273**, 1375-1383. (10.1098/rspb.2005.3458) PMC1560291 16777726

[26] Kirkpatrick M, Price T, Arnold SJ. 1990 The Darwin–Fisher theory of sexual selection in monogamous birds. Evolution **44**, 180-193. 28568205 10.1111/j.1558-5646.1990.tb04288.x

[27] Santema P, Eberhart-Hertel L, Valcu M, Kempenaers B. 2023 Data from: Sexual selection for extreme physical performance in a polygynous bird is associated with exceptional sex differences in oxygen carrying capacity. *Dryad Digital Repository*. (10.5061/dryad.pzgmsbcss) PMC10664278 37991194

